# The Effect of Toll-Like Receptor 4 in the Aqueous Humor of Endotoxin-Induced Uveitis

**DOI:** 10.3390/ijms13022110

**Published:** 2012-02-16

**Authors:** Jing Wang, Hong Lu, Yingzhi Xu, Xiaofeng Hu, Wei Chen, Shang Li

**Affiliations:** 1 Department of Ophthalmology, Chaoyang Hospital, Capital Medical University, No. 8 Baijiazhuang Road, Chaoyang District, Beijing 100020, China; E-Mails: daxi666@163.com (J.W.); xiaoting_5@126.com (Y.X.); drhuxiaofeng@gmail.com (X.H.); lishangmanager@hotmail.com (S.L.); 2 Department of Ophthalmology, Haidian Maternal and Child Health Hospital, Tongji Medical University, Beijing 100080, China; E-Mail: chenweidgj@sina.com

**Keywords:** Toll-like receptor 4, Cytokine, cytometric bead array technology, aqueous humor, Anterior Uveitis

## Abstract

In our previous study, we found that acute anterior uveitis (AAU) could be induced in wild-type mice (C3H/HeN), but it could not be induced in TLR4 gene-deficient mice (C3H/HeJ), we concluded that the translocation of transcription factor nuclear factor-κB (NF-κB) may play an important role. In this study, we examined the concentration of different cytokines in the aqueous humor of C3H/HeN mice and C3H/HeJ mice with the aim of exploring the role of different cytokines in the lipopolysaccharide (LPS) and TLR4-mediated signal transduction in the development of AAU.

## 1. Introduction

Acute anterior uveitis (AAU) is the most common form of uveitis. In the past few years, inflammation has been recognized as a major driving force of AAU. It is now well established that, starting from the initial lesion to the iris and the aqueous humor in the eye, numerous cellular and molecular inflammatory components participate in the disease process. Monocyte-derived macrophages and T-lymphocytes are the predominant invading immune cells found in evolving lesions. Both cell types produce a wide array of soluble inflammatory mediators (cytokines and chemokines) that are critically important in the initiation and perpetuation of the disease [1]. Extensive clinical and experimental evidence supports the role of a particular Gram-negative bacterium, such as Klebsiella, Salmonella, Yersinia, and Shigella or its lipopolysaccharide (LPS), in the pathogenesis of immune-mediated AAU [2]. Toll like receptors (TLRs) are a family of pattern-recognition receptors of innate immunity that recognize unique molecular signatures of microbes, known as pathogen-associated molecular patterns (PAMPs) [3–5]. TLRs are the first line of host defense and TLR activation, by their respective PAMPs, which result in proinflammatory cytokine cascades and the induction of both innate and adaptive immune responses [5,6].

Recently, our research group have demonstrated a higher expression of TLR4 on uvea-resident tissue macrophages in endotoxin-induced uveitis (EIU) than is found in normal rats [7], and C3H/HeN mice could be induced with EIU, but C3H/HeJ mice could not [8]. In this study, we compared the concentration of TNF-α, IL-1, IL-6, IL-10, and IFN-γ in the aqueous humor with EIU in C3H/HeN and C3H/HeJ mice, and inferred that TLR4 signal transduction was potentially associated with the pathogenesis of EIU and that cytokines could be used as indicators for early detection of the disease.

## 2. Results

### 2.1. Concentration of Tumor Necrosis Factor-α in Aqueous Humor

**Figure 1:**
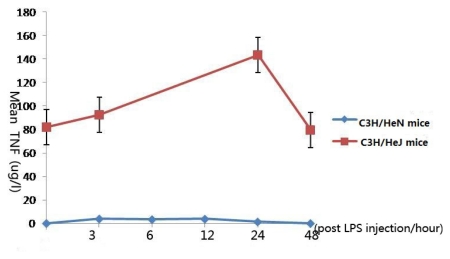
The expression levels of tumor necrosis factor-α in the aqueous humor at different time points (expressed as the mean μg/L ± SD). The concentration of TNF-α in the aqueous humor of C3H/HeN mice reached a peak 3 h after injection, then gradually decreased 12 h after injection, the concentration reached the second peak. After 48 h, TNF-α concentration in the aqueous humor returned to their original levels. The concentration of TNF-α in the aqueous humor of C3H/HeJ mice reached a peak 24 h after injection, then gradually decreased.

After injection of LPS, the concentration of TNF-α in the aqueous humor of C3H/HeN mice rapidly increased, reaching a peak 3 h after injection, which was found to be statistically significant in comparison with the control group (*p* < 0.001), then gradually decreased. Twelve hours after injection, the concentration reached the second peak. There was no significant difference between 3 h group and 12 h group (*p* = 0.410). After 48 h, TNF-α concentration in the aqueous humor returned to their original levels. The original concentration of TNF-α was higher in C3H/HeJ than that in C3H/HeN mice. After injection of LPS, the concentration gradually increased, reaching a peak 24 h after injection, which was found to be statistically significant in comparison with the control group (*p* < 0.001), then gradually decreased (Figure 1).

### 2.2. Concentration of Interleukin-1 in Aqueous Humor

**Figure 2:**
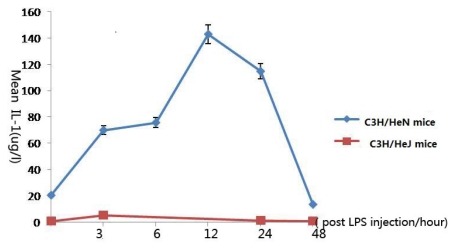
The expression levels of interleukin-1 in the aqueous humor at different time points (expressed as the mean μg/L ± SD). The concentration of IL-1 in the aqueous humor of C3H/HeN mice increased significantly 3 h after injection, reached a peak at 12 h after injection, then gradually decreased, and returned to its original concentration 48 h after injection. The concentration of IL-1 in the aqueous humor of C3H/HeJ mice reached a peak at 3 h after injection, then gradually decreased, and returned to its original concentration 48 h after injection.

In C3H/HeN mice, upon injection with endotoxin, IL-1 levels changed dramatically in the aqueous humor. Compared with the control group, the concentration of IL-1 in the aqueous humor had increased significantly 3 h after injection (*p* < 0.001), reached a peak at 12 h after injection (*p* < 0.001), then gradually decreased. It had finally returned to its original concentration at 48 h (*p* = 0.293). There was no significant difference of original concentration of IL-1 between C3H/HeN mice and C_3_H/HeJ mice (*p* = 0.637). After injection of LPS, the concentration of IL-1 in C3H/HeJ mice increased, but its peak concentration was still much lower than the concentration of C3H/HeN mice (Figure 2).

### 2.3. Concentration of Interleukin-6 in Aqueous Humor

**Figure 3:**
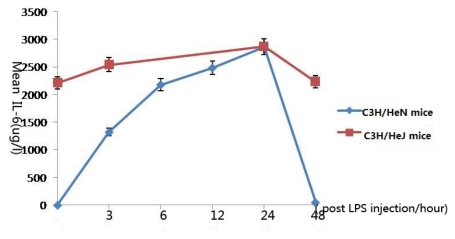
The expression levels of interleukin-6 in the aqueous humor at different time points (expressed as the mean μg/L ± SD). The concentration of IL-6 in the aqueous humor of C3H/HeN mice reached a peak 24 h after injection, then gradually decreased. The concentration of IL-6 in the aqueous humor of C3H/HeJ mice reached a peak 24 h after injection, then gradually decreased.

Notably, the concentration of IL-6 in the aqueous humor in both groups of mice reached the peak after 24 h injection, and there was no significant difference between them (*p* = 0.451). Inconceivably, the level of the concentration of IL-6 in C3H/HeJ mice was much higher than that in C3H/HeN mice (Figure 3).

### 2.4. Concentration of Interleukin-10 in Aqueous Humor

**Figure 4:**
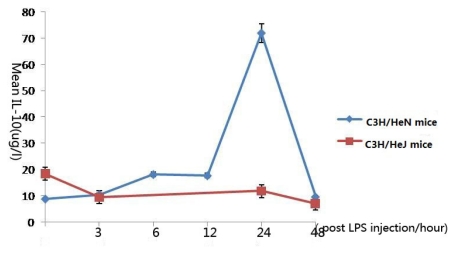
The expression levels of interleukin-10 in the aqueous humor at different time points (expressed as the mean μg/L ± SD). In C3H/HeN mice, IL-10 reached a peak concentration 24 h after injection, and returned to its original concentration 48 h after injection. The peak concentration of interleukin-10 in C3H/HeJ mice is the control group; after LPS injection, the concentration decreased gradually, but 24 h after injection, the concentration reached its second peak, then decreased.

In C3H/HeN mice, IL-10 reached a peak concentration 24 h after injection, which was found to be statistically significant in comparison with the control group (*p* < 0.001), and returned to its original concentration 48 h after injection (*p* = 0.736). Surprisingly, in C3H/HeJ mice, the peak concentration was the concentration of the control group. After LPS injection, the concentration decreased gradually, but 24 h after injection, the concentration reached its second peak, then decreased (Figure 4).

### 2.5. Concentration of Interferon-γ in Aqueous Humor

**Figure 5:**
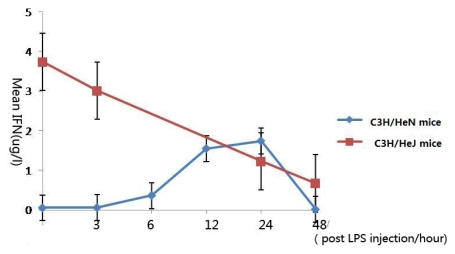
The expression levels of interferon-γ in the aqueous humor at different time points (expressed as the mean μg/L ± SD). In C3H/HeN mice, IFN-γ reached a peak concentration 24 h after injection, and returned to its original concentration at 48 h. The peak concentration of IFN-γ of C3H/HeJ mice was the original concentration, and the concentration decreased with LPS injection.

In C3H/HeN mice, IFN-γ reached a peak concentration 24 h after injection, which was found to be statistically significant in comparison with the control group (*p* < 0.001), and returned to its original concentration at 48 h (*p* = 0.499). In contrast, in C3H/HeJ mice, the peak concentration was the original concentration, and the concentration decreased with LPS injection (Figure 5).

## 3. Discussion

Recent research, in particular by Chang [9] and Lu Hong’s research group suggests that TLR4 signaling pathways might be involved in the pathogenesis of the disease [7,8,10,11]. TLR4 expression has been demonstrated on macrophages, peripheral blood monocytes, dendritic cells (DCs), and dermal vascular endothelial cells as well as in various tissues [12–15]. Lu hong’s research group demonstrated that the expression of TLR4 in the iris macrophages is up-regulated during EIU, and the TLR4 + macrophages are located in the perivascular locations, suggesting that such cells are optimally positioned to assess and to respond to LPS of invasive organisms breaking the blood-ocular barrier. Activation of TLR4 on macrophages by LPS results in the activation of the transcriptional factor, nuclear factor-κB (NF-κB), via an immunostimulatory intracellular signaling pathway. Consequently, the induction of various proinflammatory cytokines, chemokines, and antimicrobial activities initiates a rapid inflammatory response characterized by the recruitment of leukocytes to the site of infection to eliminate the invading pathogen [8]. To further elucidate the role of TLR4 in AAU, we compared the different levels of cytokines in wild-type C3H/HeN and TLR4 gene-deficient C3H/HeJ mice in this work.

EIU is a well-established experimental model [7,8,10,11], an injection of LPS to certain susceptible strains of rodents induces an acute and preferential inflammation of the iris and ciliary body that closely resembles AAU in humans. In our previous studies, we found that EIU was successfully induced in C3H/HeN mice by intraperitoneal injection of 200 μg *Vibrio cholerae* LPS. The inflammatory reaction reached a peak 24 h after injection, and subsided after 48 h [10]. The aim of this study is provide novel evidence of changes in cytokine concentrations in the aqueous humor of C3H/HeN mice and C3H/HeJ mice over the course of AAU.

TNF-α reached a peak concentration in the early stages of inflammation; thus, we suggested TNF-α 6might play an important role in the initiation of the inflammatory response. Perez-Guijo [16] and Santos-Lacomba [17] reported that the concentration of TNF-α in the aqueous humor of uveitis patients were significantly higher than that of a normal control group. Taken together, the evidence indicates that TNF-α plays an important role in the etiology of uveitis. The concentration of TNF-α reached the second peak 12 h after injection, maybe there are two reasons for this: first, TNF-α is the Th1 cytokine produced mainly by macrophage. In our previous study, we found macrophage was in the activated status 12 h after LPS stimulation [8], maybe the secretion is correlated with the activation; second, cytokines is a net, and the secretion of one cytokine may be influenced by the other cytokines.

In C3H/HeJ mice, the concentration of TNF-α was so much higher than that in C3H/HeN mice, may be the concentration levels could affect its role in inflammation. In C3H/HeN mice, IL-1 reached its peak 12 h after LPS injection. Similar with TNF-α, the main secretory cell is macrophage. We presumed that TNF-α is the cytokine in the early stage of the inflammation, IL-1 is the cytokine in other stage of the inflammation. Furthermore, IL-1 and TNF-α reached the peak at the same time, maybe the secretion of them was influenced by each other. In C3H/HeJ mice, IL-1 reached the peak concentration 3 h after LPS injection, and the concentration was much lower than that in C3H/HeN mice. We supposed the concentration level was important for cytokine to play its role.

Twenty four hours after LPS injection, the inflammation of C3H/HeN mice reached its peak, and IL-6 reached its peak. For C3H/HeJ mice, the concentration of IL-6 was higher than that in C3H/HeN mice; maybe IL-6 is not a pro-inflammatory cytokine in gene-deficient mice. The original concentration of IL-6 was very high, so we suggest the secretion of IL-6 was affected by other cytokine and the role have changed.

In C3H/HeN mice, IL-10 reached its peak 24 h after LPS injection, but the concentration peak in C3H/HeJ mice was the original concentration. Schreiber T *et al*. reported that IL-10 is not a strictly Th2 cytokine, but it shares many features with them, and it was thought to act as a “deactivator” of macrophage [18]. In our study, we found that the peak of inflammation was 24 h after LPS injection, then the inflammation reaction decreased. So the cytokine concentration was consistent with the clinical manifestations.

IFN-γ, together with TNF-α, is Th1 cytokines. In C3H/HeN mice, TNF-α played the role in the early stage of the inflammation, IFN-γ may play the role in the fastigium of AAU. Similar with the tendency of IL-10, in C3H/HeJ mice, the concentration peak of IFN-γ was the original concentration, we presumed that because of gene defect, LPS-TLR-4- NF-κB could not be activated [8], and the secretion of the cytokine was changed.

EIU is closely related to inflammatory cytokines produced by Thl-type cellular immune responses, such as IL-l, TNF-α, and IFN-γ. An excessive Thl response could lead to contact hypersensitivity and cell-mediated autoimmune responses [19]. Some inhibitory cytokines such as IL-10 could reduce pathological damage to eye tissue. Now, lots of biological agents have been applied in therapy of uveitis [20,21]. Further investigation into the interaction and dynamic changes of cytokines may provide insights into the pathogenesis of EIU, potentially leading to more-effective treatments.

## 4. Experimental Section

### 4.1. LPS

Lipopolysaccharide (*V. cholera*, classical Biotype, serotype Ogawa) was kindly provided by Lanzhou Institute of Biologic Products (Lanzhou, China). Endotoxin-induced uveitis was induced as previously described [8]. Mice received a single injection of 200 μg LPS dissolved in 100 μL sterilesaline (NS)

### 4.2. Animals

Sixty C3H/HeN male mice, 6 to 8 weeks old and weighing between 16 g and 25 g, were obtained from the Beijing Wei Tong Li Hua Laboratory (Animal Victoria Limited, Beijing, China) and housed in 12 h light/12 h dark cycles. The mice were randomly divided into an experimental group (*n* = 50) and a control group (*n* = 10). The experimental group was further randomly divided into five subgroups of 10 mice each, according to post-LPS injection time points (3 h, 6 h, 12 h, 24 h and 48 h). Forty C3H/HeJ male mice, 6 to 8 weeks old and weighing between 16 g and 25 g, were obtained from Model Animal Research Center (Nanjing, China) and housed in 12 h light/12 h dark cycles. The mice were randomly divided into an experimental group (*n* = 30) and a control group (*n* = 10). The experimental group was randomly divided into three subgroups of 10 mice each, according to post-LPS injection time points (3 h, 24 h and 48 h, the key point time of the development of inflammation according to our previous research [8]). Throughout this study, all procedures adhered to the Institute for Laboratory Animal Research guidelines (Guide for the Care and Use of Laboratory Animals).

### 4.3. Apparatus Used in Cytometric Bead Array

BD™ Flow Cytometry (FACSCalibur, BD Company, San Diego, CA). BD™ Cell Quest and CBA software (material number 550065; BD Company) BD™ CBA Mouse TNF, IL-1, IL-6, IL-10, and IFN-γ Flex Set (material number 558266; BD Company). The BD FACSCalibur used is an automated, multicolour, bench top flow cytometer. It uses BD software for data analysis.

### 4.4. Sample Collection

The aqueous humor samples were collected using a microinjector. Ten samples were pooled together and replicated performed for analysis. Then samples were centrifuged at 705× *g*, at 4 °C, for 3 min. The supernatant was collected and stored at −80 °C.

### 4.5. Generation of Standard Curves

The cytokine standard solutions included with the BD CBA Flex Sets were serially diluted to the following concentrations: 2500 μg/L, 1250 μg/L, 625 μg/L, 312 μg/L, 156 μg/L, 80 μg/L, 40 μg/L, 20 μg/L, and 0 μg/L. A control sample was also included in the measurements. Measurements for the different concentrations were obtained using the FACSCalibur, and standard curves were generated using the BD CBA software.

### 4.6. Sample Preparation and Analysis

A total of 50 μL of sample was mixed with 50 μL of capture beads and 50 μL of PE-labeled detection antibodies. After incubation in the dark at room temperature for 2 h, 1 mL of soluble protein master buffer lotion was added. The sample was then centrifuged at 200× g for 5 min and washed twice, before the addition of 300 μL buffer lotion. Results were obtained using BD CellQuest and BD CBA software.

### 4.7. Statistics

Quantitative data were analyzed with one-way analysis of variance (ANOVA) followed by Significant Difference Procedure (LSD) test for multiple comparisons among experimental groups with control groups. Data was performed by SPSS (version 13.0; SPSS Inc., Chicago, IL) statistical software. A *p* value of 0.05 or less was interpreted as indicating statistical significance when comparing experimental and control groups.

## 5. Conclusions

The cytokine levels in the aqueous humor of C3H/HeN mice were consistent with what would be expected during the TLR4 signal transduction related inflammation process. Further investigation into the interaction and dynamic changes of cytokines may provide insights into the pathogenesis of AAU, potentially leading to more effective treatments.
